# Predicting lncRNA-disease associations based on heterogeneous graph convolutional generative adversarial network

**DOI:** 10.1371/journal.pcbi.1011634

**Published:** 2023-11-29

**Authors:** Zhonghao Lu, Hua Zhong, Lin Tang, Jing Luo, Wei Zhou, Lin Liu

**Affiliations:** 1 School of Information, Yunnan Normal University, Yunnan, People’s Republic of China; 2 Key Laboratory of Educational Information for Nationalities Ministry of Education, Yunnan Normal University, Yunnan, People’s Republic of China; 3 State Key Laboratory for Conservation and Utilization of Bio-resource in Yunnan, School of Life Sciences and School of Ecology and Environment, Yunnan University, Kunming, People’s Republic of China; 4 School of Software, Yunnan University, Kunming, People’s Republic of China; Ecole Polytechnique Federale de Lausanne, SWITZERLAND

## Abstract

There is a growing body of evidence indicating the crucial roles that long non-coding RNAs (lncRNAs) play in the development and progression of various diseases, including cancers, cardiovascular diseases, and neurological disorders. However, accurately predicting potential lncRNA-disease associations remains a challenge, as existing methods have limitations in extracting heterogeneous association information and handling sparse and unbalanced data. To address these issues, we propose a novel computational method, called HGC-GAN, which combines heterogeneous graph convolutional neural networks (GCN) and generative adversarial networks (GAN) to predict potential lncRNA-disease associations. Specifically, we construct a lncRNA-miRNA-disease heterogeneous network by integrating multiple association data and sequence information. The GCN-based generator is then employed to aggregate neighbor information of nodes and obtain node embeddings, which are used to predict lncRNA-disease associations. Meanwhile, the GAN-based discriminator is trained to distinguish between real and fake lncRNA-disease associations generated by the generator, enabling the generator to improve its ability to generate accurate lncRNA-disease associations gradually. Our experimental results demonstrate that HGC-GAN performs better in predicting potential lncRNA-disease associations, with AUC and AUPR values of 0.9591 and 0.9606, respectively, under 10-fold cross-validation. Moreover, our case study further confirms the effectiveness of HGC-GAN in predicting potential lncRNA-disease associations, even for novel lncRNAs without any known lncRNA-disease associations. Overall, our proposed method HGC-GAN provides a promising approach to predict potential lncRNA-disease associations and may have important implications for disease diagnosis, treatment, and drug development.

## Author summary

Long non-coding RNAs (lncRNAs) refer to non-coding RNAs with lengths exceeding 200 nucleotides, and they play pivotal roles in various diseases, including cancer, cardiovascular, and neurological disorders. However, accurately predicting their associations with diseases presents a formidable challenge, particularly given the limitations of existing methods in handling complex and diverse data as well as sparse information. To address these issues, we introduce a novel computational approach known as HGC-GAN. This method combines the robust capabilities of heterogeneous graph convolutional neural networks (GCN) and generative adversarial networks (GAN) to predict potential associations between lncRNAs and diseases. Leveraging rich biological information, it overcomes challenges related to data scarcity and the absence of negative samples. Our empirical results demonstrate that HGC-GAN outperforms existing models and achieves impressive results in predicting the associations between lncRNAs and diseases under rigorous cross-validation. Notably, HGC-GAN has proven its reliability across datasets of varying scales. Furthermore, our case studies illustrate its ability to predict associations of novel lncRNAs with diseases that have no known links. We believe that HGC-GAN holds great promise in advancing disease diagnosis, treatment, and drug development.

## Introduction

Long non-coding RNA (lncRNA) is a non-coding RNA with a length of over 200 nucleotides and is closely related to various human diseases, including cancers and cardiovascular and nervous system disorders [1,2,3]. Despite its significance, the number of lncRNA-disease associations verified by biological experiments remains limited. Accurate prediction of potential lncRNA-disease associations using computational methods can effectively reduce experimental costs and workloads while improving the efficiency of biological experiments. Consequently, it is crucial to develop effective prediction models that can analyze multi-modal biological data to accurately predict potential lncRNA-disease associations.

In recent years, numerous computational models have been proposed, which can be broadly classified into two categories: biological network-based methods and deep learning-based methods. The former leverages known associations between lncRNAs, diseases, and other biological entities to construct multi-source biological networks, which are then used to predict potential lncRNA-disease associations using various computational approaches [4,5,6,7]. However, these methods have some limitations, such as their inability to predict new lncRNAs and the incomplete and inaccurate representation of biomolecular interactions.

Deep learning-based approaches typically employ neural networks to generate low-dimensional representations from high-dimensional features of lncRNAs and diseases and use classifiers to predict potential lncRNA-disease associations [8,9,10,11]. Additionally, numerous graph neural network-based models rely solely on a single lncRNA-disease association data and overlook the importance of incorporating lncRNA sequence extraction features. Recent studies have highlighted that integrating multi-source data can significantly enhance predictive performance [12,13,14]. Although these methods offer some advantages, sparse association information and the absence of negative samples during training may reduce the reliability of prediction results.

To address these challenges, this study proposes a deep learning model based on heterogeneous graph convolutional neural networks and generative adversarial networks (HGC-GAN) for predicting potential lncRNA-disease associations. HGC-GAN integrates the strengths of heterogeneous graph convolutional neural networks (HGCN), which can effectively aggregate biological entity associations and embed them into node features, and generative adversarial networks (GAN), which can automatically generate low-cost samples without repeated sampling.

HGC-GAN constructs a lncRNA-miRNA-disease heterogeneous network (LMDN) by integrating different biological association data. The generative network consists of HGCN and G-Network, which generate node embeddings by aggregating multi-source association information and higher-order neighbor information. The discriminator then distinguishes between real and fake associations, continuously improving the performance of predicting potential lncRNA-disease associations through adversarial training.

Experimental results demonstrate that HGC-GAN outperforms state-of-the-art models under 10-fold cross-validation and exhibits excellent robustness for datasets of varying sizes. Additionally, a case study confirms HGC-GAN’s effectiveness in predicting potential lncRNA-disease associations, including its ability to predict potential associations for novel lncRNAs without known associations.

In summary, this study presents HGC-GAN, an innovative model that combines HGCN and GAN to predict potential lncRNA-disease associations. HGC-GAN capitalizes on the information richness of biomolecules while addressing the challenges of sparse association information and the absence of negative samples. The code and dataset for HGC-GAN are available at https://github.com/ZhonghaoLu/HGC-GAN.

## Materials and methods

### Datasets

Two datasets were constructed by integrating various types of biological association data. The association data used in this study have been demonstrated to have a direct impact on the occurrence and progression of diseases, as confirmed by scientific research. In Dataset 1, a portion of the data utilized originates from the lncRNA-disease association prediction study conducted by Li et al. [15]. This dataset was supplemented with lncRNA-miRNA association data from the starBaseV2.0 database [16]. Dataset 1 comprises 861 lncRNAs, 673 miRNAs, 432 diseases, 4518 lncRNA-disease associations, 4189 miRNA-disease associations, and 2105 lncRNA-miRNA associations. In Dataset 2, sequence data for 1363 lncRNAs were obtained from the NCBI database [17]. lncRNA-disease and lncRNA-miRNA association data were extracted from LncRNADiseaseV2.0 [18,19], Lnc2CancerV3.0 [20], and starBaseV2.0 [16] databases. MiRNA-disease association data were acquired from HMDDV3.2 [21]. After duplicate removal, Dataset2 includes 1363 lncRNAs, 1190 miRNAs, 501 diseases, 5338 lncRNA-disease associations, 6763 miRNA-disease associations, and 2291 lncRNA-miRNA associations. Dataset 3 was derived from the dataset used by Zhou et al. [22] and included 2697 lncRNA-disease associations, 1002 lncRNA-miRNA associations, and 13562 miRNA-disease associations. Dataset 4 is from the dataset used in reference Zhang et al. [23] and contains 1151 lncRNA-disease associations, 10102 lncRNA-miRNA associations, and 4634 miRNA-disease associations.

Unless otherwise specified, all experiments were conducted using Dataset 1, while Dataset 2 was employed in case studies for predicting potential associations for new lncRNAs. The statistics of the datasets are presented in Table 1. Eq (1) was also used to represent the sparsity and size of the different datasets.


$$LDA\;sparsity=\frac{count\;one\;elements}{total\;elements}\times 100\%$$ (1)

**Table 1:** The statistics of datasets.

Dataset	lncRNA	disease	miRNA	LDA	MDA	LMA	LDA sparsity
Dataset1	861	432	673	4518	4189	2105	0.012
Dataset2	1363	501	1190	5338	6763	2291	0.008
Dataset3	240	412	495	2697	13562	1002	0.027
Dataset4	1723	236	675	1151	4634	10102	0.003

### HGC-GAN

**Fig 1:**
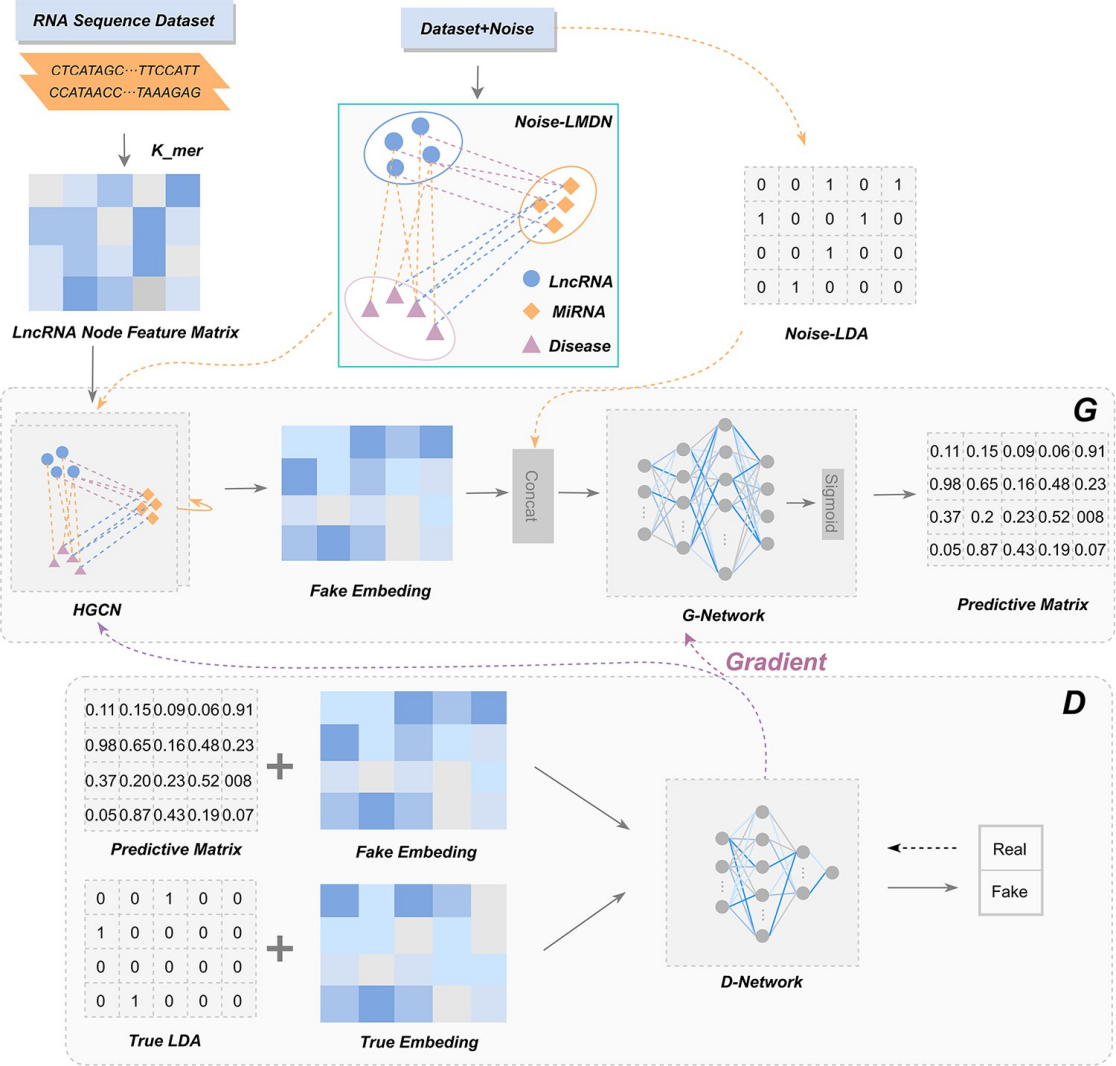
The diagram of HGC-GAN.

The integration of HGCN and GAN within the HGC-GAN framework not only addresses data sparsity but also effectively obtains entity embeddings from multi-source associated neighbor information. By aggregating this multi-source neighbor information, the generator acquires embedded representations of lncRNAs and diseases, which are then fed into the G-Network to compute the corresponding association scores. By processing both real and fake data, the discriminative ability of the discriminator is continuously updated and subsequently fed back to the generator to enhance its prediction performance. Fig 1 illustrates the architecture of our HGC-GAN model.

### Data preprocessing

#### Construct heterogeneous association network

First, three association matrices, lncRNA-disease (A), miRNA-disease (B), and lncRNA-miRNA (C) were constructed. Taking the lncRNA-disease association matrix (A) as an example, if lncRNA L is associated with disease D in the association data, the corresponding element value in the association matrix A is 1; otherwise, it is 0. The authenticity of known associations is ensured as the collected data comes from publicly available datasets, and lncRNAs have been demonstrated in numerous biological studies to have significant relationships with certain human diseases.


$$R_{l\times d}(L,D)=\left\{ \begin{array}{l} 1,If\;L\;and\;D\;have\;association\\ 0,Otherwise \end{array}\right.$$ (2)

**Fig 2:**
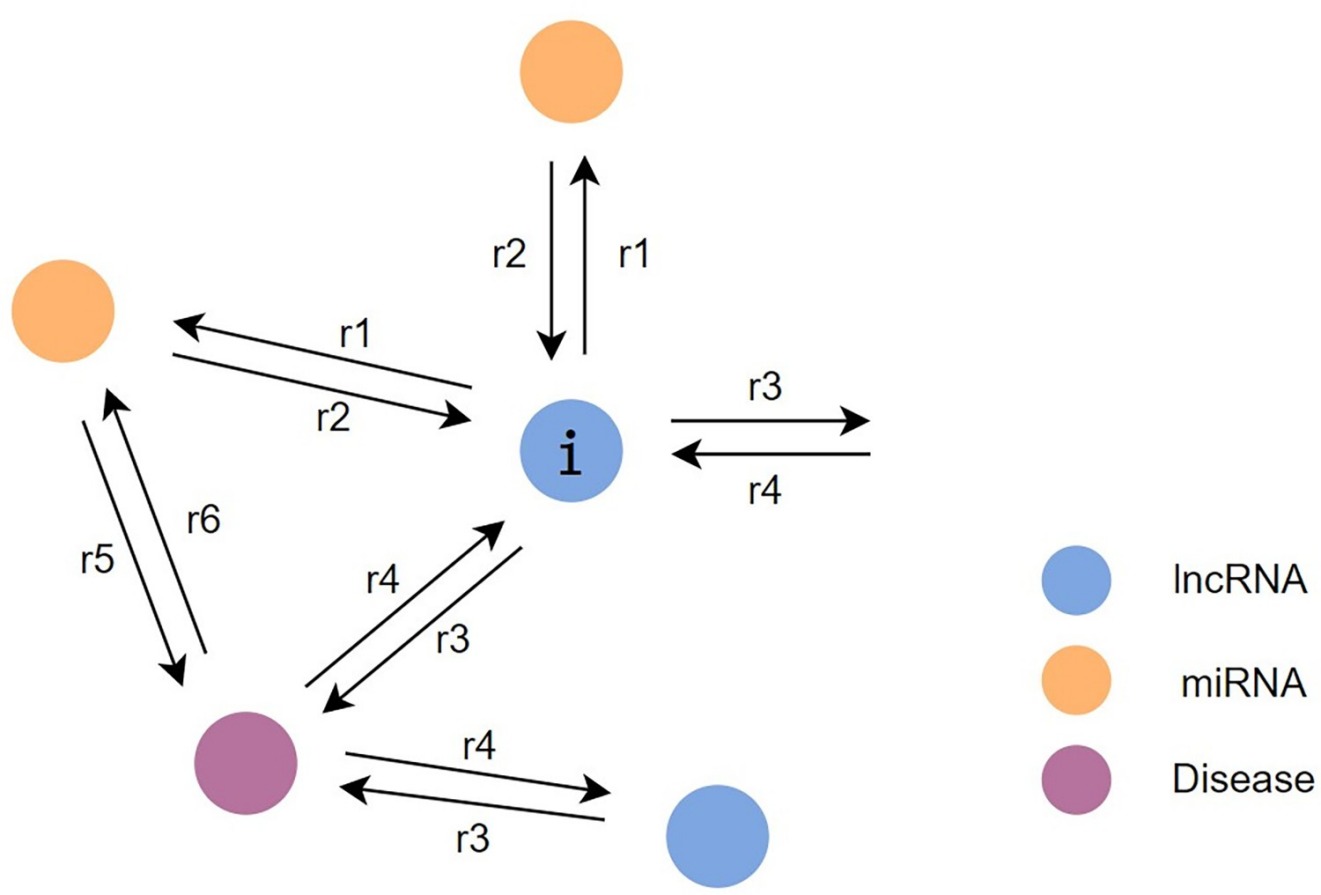
Node aggregation.

In the experiments, the dimension of A is m x n, with other matrices exhibiting similar dimensions. After constructing the association matrix, the lncRNA-disease-miRNA heterogeneous network (LMDN) was constructed. Within the LMDN, there are three types of nodes (lncRNA, miRNA, disease), and each type has multiple nodes. For instance, there are numerous distinct lncRNAs within the lncRNA node type. If a particular lncRNA and miRNA are associated, the constructed heterogeneous graph will have two directed edges connecting these nodes—one from the miRNA to the lncRNA, and another from the lncRNA to the miRNA, representing the connection between the nodes. Consequently, there will be two directed edges between every pair of associated nodes, as shown in Fig 2. Ultimately, LMDN is a sparse heterogeneous network consisting of 1966 nodes and 10812 edges.

#### Extract sequence feature by K_mer

**Fig 3:**
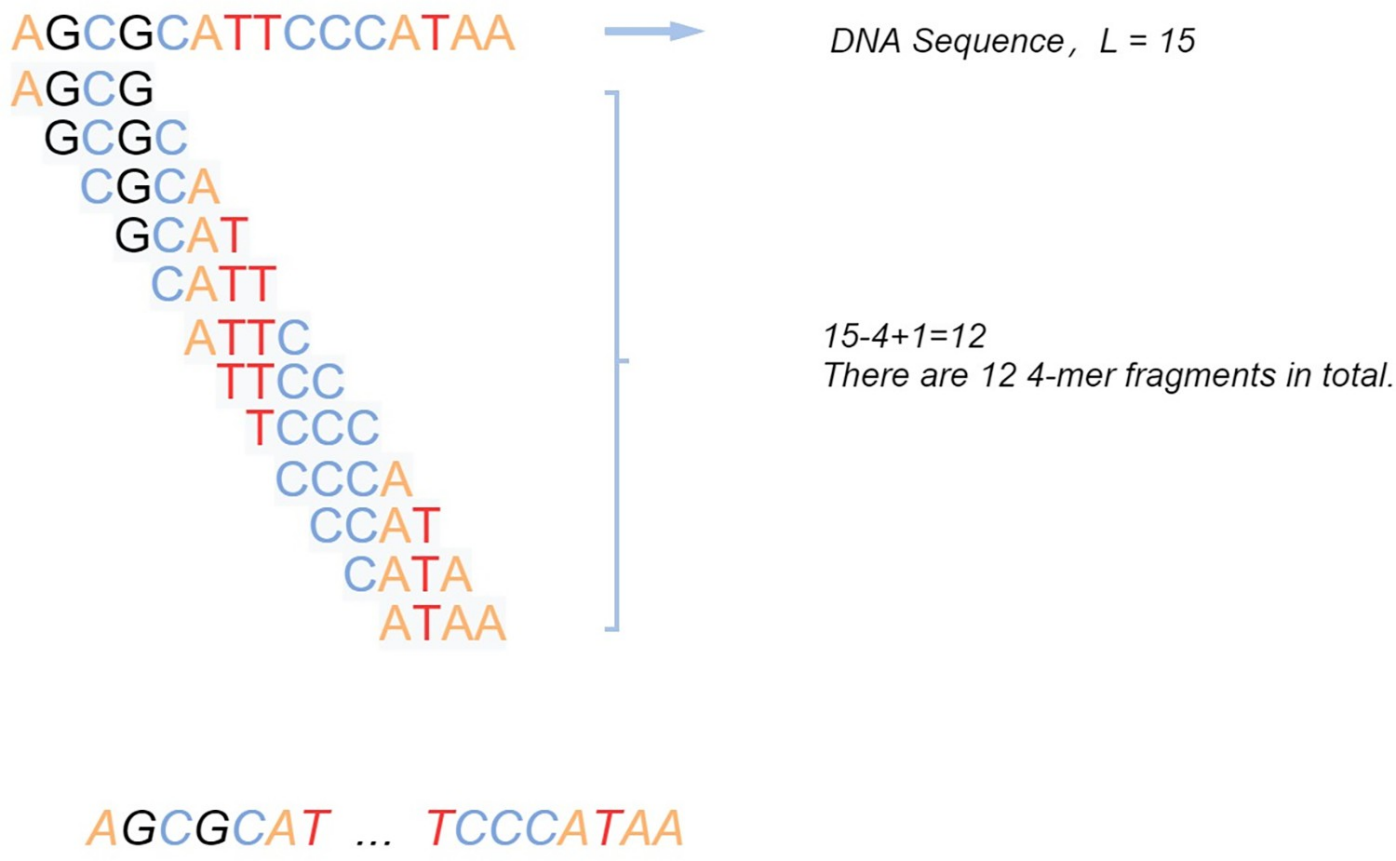
K_mer.

In Dataset 2, the sequence information of lncRNAs was collected, and the K-mer method was employed to extract features from the sequences as the initial features of lncRNAs. This approach was then applied to predict potential associations between new lncRNAs with no known lncRNA-disease associations and diseases. K-mer refers to iteratively selecting base fragments of K nucleotide units from one end of a continuous nucleotide sequence. For example, in a DNA sequence, a sequence composed of N bases would typically have *N*−*K*+1 K-mers, with a total of 4^*k*^ possible K-mers. This is illustrated in Fig 3.

The frequency information of K-mers refers to the number of occurrences of a particular K-mer fragment within the DNA sequence. Consequently, each lncRNA sequence can be represented as a numerical vector composed of K-mer frequencies, with a dimension of 4^K. The obtained numerical vectors serve as the initial features of lncRNA nodes. The initial features of lncRNA L can be represented as:

$$L=[L_{1},L_{2},L_{3},\ldots ,L_{i},{\ldots ,L}_{4^{k}}]$$ (3)

*L*_*i*_ (*i* = 1,2,3,…,4^*k*^) denotes the frequency of occurrence of the i-th K-mer fragment in the sequence.

### Generative adversarial networks

HGC-GAN employs the generative adversarial network framework. Through adversarial training, the generator produces data that more closely approximates the real data distribution, while the discriminator’s capacity to discern real and fake data incrementally improves. Ultimately, the generator’s predictive performance is continuously enhanced. The objective function is presented below.


$${min}_{G}{max}_{D}V(G,D)=E_{i∼p_{r}}log(D(i))+E_{z∼p_{z}}log(1-D(G(Z)))$$ (4)

*G* denotes the generator; *D* denotes the discriminator; *P*_*r*_ indicates the real lncRNA-disease association distribution; *P*_*z*_ represents noise distribution; *i* represents *lncRNA*_*i*_ vector subject to real association distribution; *z* represents the vector obeying the noise distribution.

### Generator of HGC-GAN

The generator in Fig 1, Part G, comprises the Heterogeneous Graph Convolutional Network (HGCN) and G-network. HGCN takes noise, LMDN, and initial features as inputs. To generate fake associations, we add fake edges to the lncRNA-disease (A) and lncRNA-miRNA (C) association matrices, creating noise association matrices A’ and C’. The noise-containing heterogeneous network (Noise-LMDN) is constructed using A’ and C’, and combined with the true association matrix A to form the complete LMDN.

By introducing noise associations and constructing the Noise-LMDN, we enhance the generator’s performance by encouraging it to produce more realistic fake associations.

#### HGCN

In the heterogeneous graph Noise-LMDN, each association is processed separately, allowing information from source nodes to be passed to target nodes along different associations. Features for the same target node are updated by aggregating information from different associations. As edges in heterogeneous networks are bidirectional, there are six associations, referred to as six-seed networks (L-D, D-L, L-M, M-L, M-D, D-M). The formula is as follows:

$$H_{dst}^{\left(l+1\right)}={AGG}_{r\in R,r_{dst}=dst}\left(f_{r}(G_{r},H_{r_{src}}^{l},H_{r_{dst}}^{l})\right)$$ (5)

*r* represents each association; *R* is the set of six associations; *scr* represents the source node; *dst* denotes the target node (node whose feature is to be updated); *l* is the convolution layer of heterogeneous graph; $H_{dst}^{\left(l+1\right)}$ represents the embedding of target node at *l*+1-th layer; $H_{r_{src}}^{l}$ denotes the embedding of source node at *l*-th layer; *G*_*r*_ represents the subnetwork; *f*_*r*_ represents the processing module corresponding to each association *r*; *AGG* is the aggregation function; *r*_*dst*_ = *dst* is used to represent the condition where the target node equals the given node *dst* in order.

As depicted in Fig 2, each node aggregates information from multi-source neighbor nodes and higher-order neighborhoods, utilizing the association information of the heterogeneous network to update node embeddings.

In each processing module of the association, a graph convolutional neural network (GCN) is employed to update the node embedding. Instead of using a shared processing module, six processing modules corresponding to the six associations are employed, i.e., six GCNs act on the corresponding associations. The way messages are aggregated for each association is the same:

$$H_{i}^{\left(l+1\right)}=\sigma (b^{\left(l\right)}+\sum_{j\in N(i)}\frac{1}{c_{ji}}H_{j}^{\left(l\right)}W^{\left(l\right)})$$ (6)

*N*(*i*) represents the set of neighbor nodes of node *i*.*j* represents a neighbor node of node *i*; *W*^(*l*)^ and *b*^(*l*)^ represent the weight and bias matrix of the convolutional neural network in layer *l* respectively; *σ* represents the activation function; $\frac{1}{c_{ji}}$ is the normalization operation:

$$c_{ji}=\sqrt{\left|N(j)\right|}\sqrt{\left|N(i)\right|}$$ (7)

*N*(*j*) and *N*(*i*) denote the out-degree and in-degree of node *j* and node *i*, respectively.

In the subgraph of each association, there are only two types of nodes. In the six association subnetworks, each node receives information from two different types of nodes. For example, in the D-L network, lncRNAs receive information from diseases, while in the M-L network, lncRNAs receive information from miRNAs. When the six association subnetworks are aggregated, each type of node has two embeddings that are obtained by aggregating information from other nodes. Then, the two embeddings are combined to obtain the final embeddings using Eq (4), which aggregates multi-source and higher-order neighbor information. The message-passing paradigm is as follows:

$$Calculation\;on\;Edge:m_{e}^{\left(t+1\right)}=\phi (x_{v}^{\left(t\right)},x_{u}^{\left(t\right)},w_{e}^{\left(t\right)}),(u,v,e)\in \varepsilon$$ (8)

$$Calculation\;at\;Point:x_{v}^{\left(t+1\right)}=\psi \left(x_{v}^{\left(t\right)},\rho (\left\{m_{e}^{\left(t+1\right)}:(u,v,e)\in \varepsilon \right\})\right)$$ (9)

*x*_*v*_ and *x*_*u*_ represents the features on node *v* and *u*; *w*_*e*_ represents the feature on the edge (*u*, *v*); *ϕ* is the message function, which generates messages by combining the features of two endpoints. *ρ* is the aggregation function, which aggregates the message received by the node; *ψ* represents the update function, which combines the updated messages and the initial features of nodes to update node embeddings.

Take the node *lncRNA*_*i*_ in Fig 2 as an example. *lncRNA*_*i*_ participates in four association processing modules *r*1, *r*2, *r*3 and *r*4, but only performs message aggregation in *r*2 and *r*4 processing modules. *lncRNA*_*i*_ receives messages from other nodes through Eq (7). Then, the graph convolution is realized by Eq (5) and the message is aggregated to obtain two embeddings *L*_*m*_ and *L*_*d*_. The embedding *L* is obtained by aggregating *L*_*m*_ and *L*_*d*_ through Eq (8), and the aggregation method is implemented by Eq (4). The final embedding *L*^(*l*+1)^ is obtained by *ψ* combining the initial features of *lncRNA*_*i*_ and *L*.

#### G-Network

The embeddings of lncRNA, miRNA and disease are obtained by HGCN. Assuming that the dimension of the obtained lncRNA embedding is *w*, then its embedding matrix is *E*_*l*×*w*_. Meanwhile, the *Noise*−*R*_*l*×*d*_ is also obtained for the input of generator. *E*_*l*×*w*_ and *Noise*−*R*_*l*×*d*_ are integrated into matrix *M* for G-Network training. G-Network consists of four layers of fully connected neural networks, which finally outputs an association prediction matrix *P* with size *l*×*d*.

#### The loss function of generator

This paper adopts the concept of Wasserstein GAN (WGAN) to address the mode collapse issue in the original generative adversarial network, which is not conducive to generating diverse samples and predicting potential lncRNA-disease associations. Moreover, to address the problems of association data sparsity and the tendency of the generator to generate all-1 data, we introduced the concept of CFGAN-ZP [24] into the loss function of the generator and added a filter matrix to prevent the degradation of model performance during training. The specific loss function is as follows:

$$Loss_{G}=-E_{i∼P_{g}}[f_{w}({\hat{r}}_{i}⊙(e_{i}+k_{i}))]+\alpha ∙\sum_{j}{\left(x_{ij}-{\hat{x}}_{ij}\right)}^{2}$$ (10)

*P*_*g*_ represents the fake association distribution generated by the generator; *f*_*w*_ represents the discriminator network whose last layer is not a nonlinear activation layer under the condition that the parameter *w* does not exceed a certain range. ${\hat{r}}_{i}⊙(e_{i}+k_{i})$ is the filter term, where ${\hat{r}}_{i}$ denotes the association score of *lncRNA*_*i*_ generated by the generator; *e*_*i*_ denotes the input noise data; *k*_*i*_ then indicates that a fraction of *lncRNA*_*i*_ that is not associated with disease is randomly selected as a negative sample and assigned a value of 1. ⊙ denotes the pairwise multiplication of two matrices; α is the hyperparameter of the regularization term; *x*_*ij*_ denotes the true association distribution of *lncRNA*_*i*_ with diseases; and ${\hat{x}}_{ij}$ denotes ${\hat{r}}_{i}⊙(e_{i}+k_{i})$, the filter term, which is also the fake data input to the discriminator network.

### Discriminator of HGC-GAN

The discriminator takes as input both fake data and real data. The fake data is obtained by integrating the association prediction matrix *P* generated by G-Network with the embedding *E*_*fake*_ obtained from HGCN. The real data is obtained by integrating the real association matrix *R*_*l*×*d*_ with the embedding *E*_*true*_ obtained from the real heterogeneous graph LMDN, which is fed into HGCN.

### D-Network

The D-Network has a similar structure to the G-Network and also consists of a four-layer fully connected neural network. Real and fake data are fed into the D-Network to obtain *l*×1 scores, respectively. The scores are then passed to the loss function of the discriminator for feedback. The loss function of the discriminator also incorporates the idea of WGAN and adds a filter term. The loss function of the discriminator is as follows, where *P*_*r*_ denotes the real association between lncRNA and disease:

$$Loss_{D}=E_{i∼P_{g}}[f_{w}({\hat{r}}_{i}⊙(e_{i}+k_{i}))]-E_{i∼P_{r}}[f_{w}(i)]$$ (11)

## Results and discussion

### Evaluation metrics

This paper uses 10-fold cross-validation to evaluate the performance of HGC-GAN. In this process, all samples are divided into 10 sub-datasets, and each sub-dataset is selected in turn as the test set, while the other nine sub-datasets are used as the training set. The final result is the average of 10 experiments. The ROC curve is used to evaluate the performance of the HGC-GAN model and can depict the relationship between the true positive rate (TPR) and false positive rate (FPR) at different thresholds. Additionally, the AUPR values are calculated to assess the overall performance of the model in the presence of extreme imbalance between positive and negative samples. The definitions of TPR, FPR, recall, and precision are as follows:

$$TPR=\frac{TP}{TP+FN}=Recall$$ (12)

$$FPR=\frac{FP}{FP+TN}$$ (13)

$$Precision=\frac{TP}{TP+FP}$$ (14)

*TP* is the number of positive samples that the classifier correctly classified as positive samples. *TN* is the number of negative samples correctly classified as negative by the classifier. *FP* is the number of negative samples that the classifier incorrectly classified as positive samples, and *FN* is the number of positive samples that the classifier incorrectly classified as negative samples.

**Fig 4:**
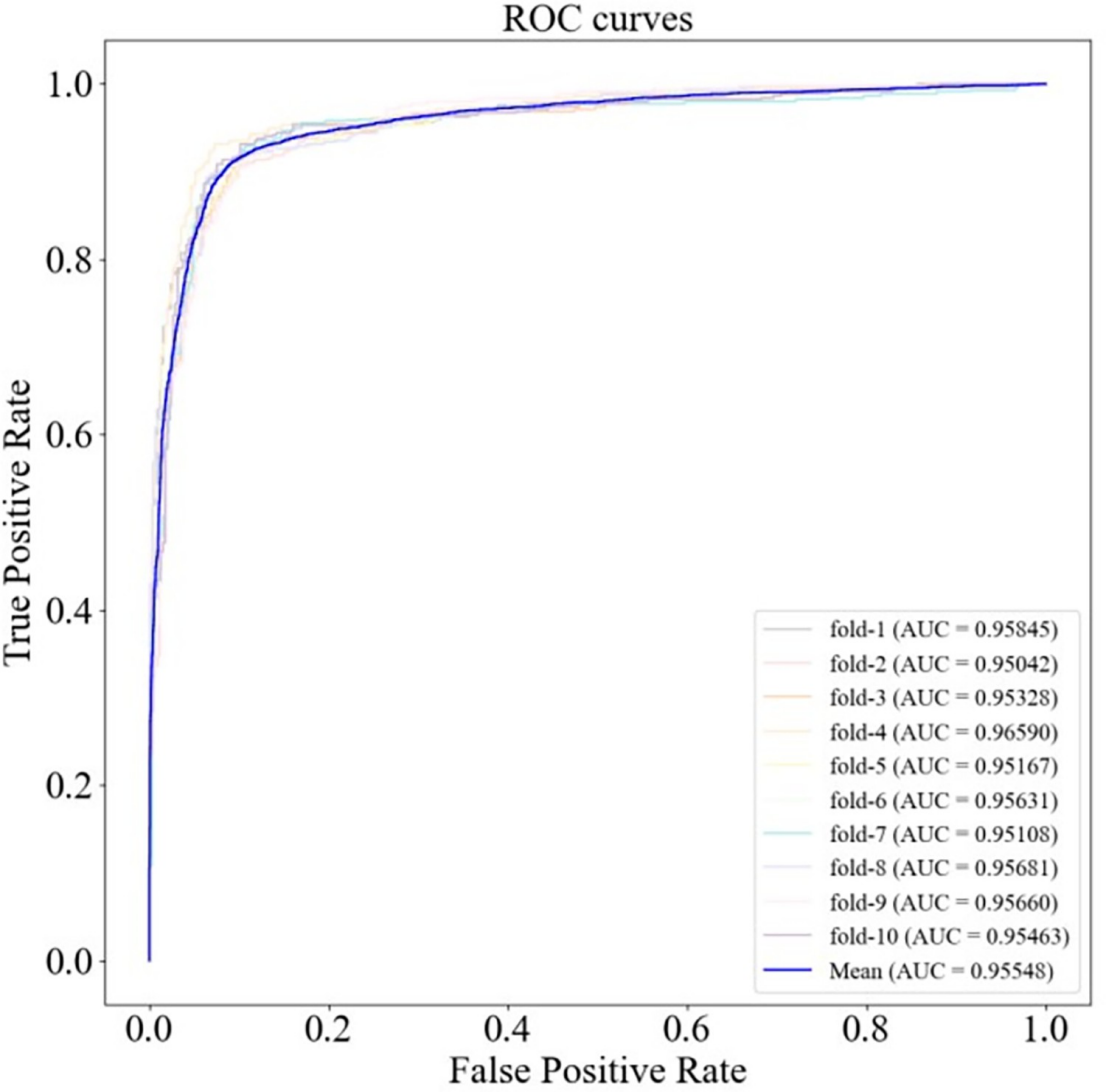
ROC curve under 10-fold cross-validation.

**Fig 5:**
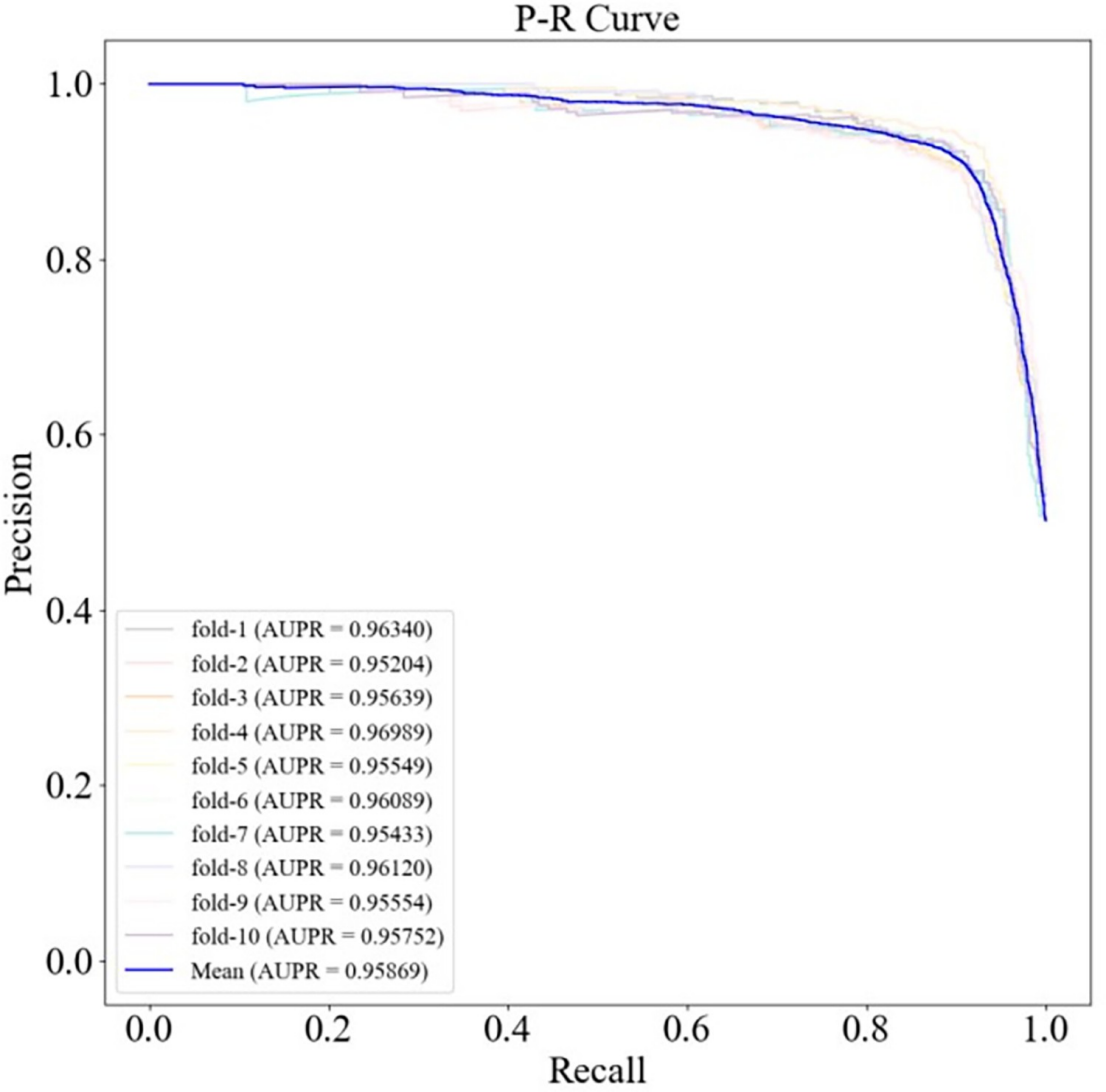
P-R curves under 10-fold cross-validation.

**Fig 6:**
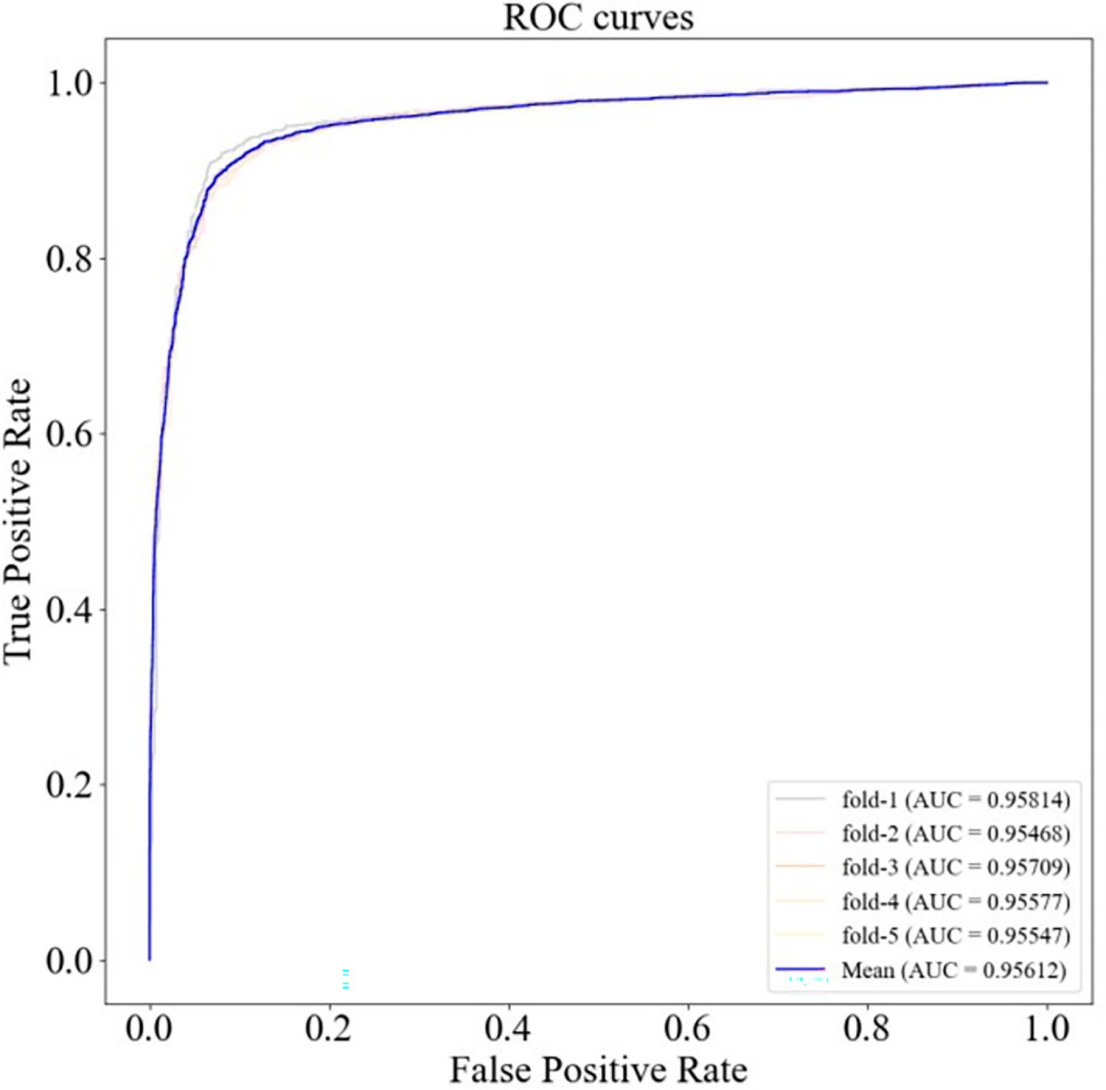
ROC curve under 5-fold cross-validation.

**Fig 7:**
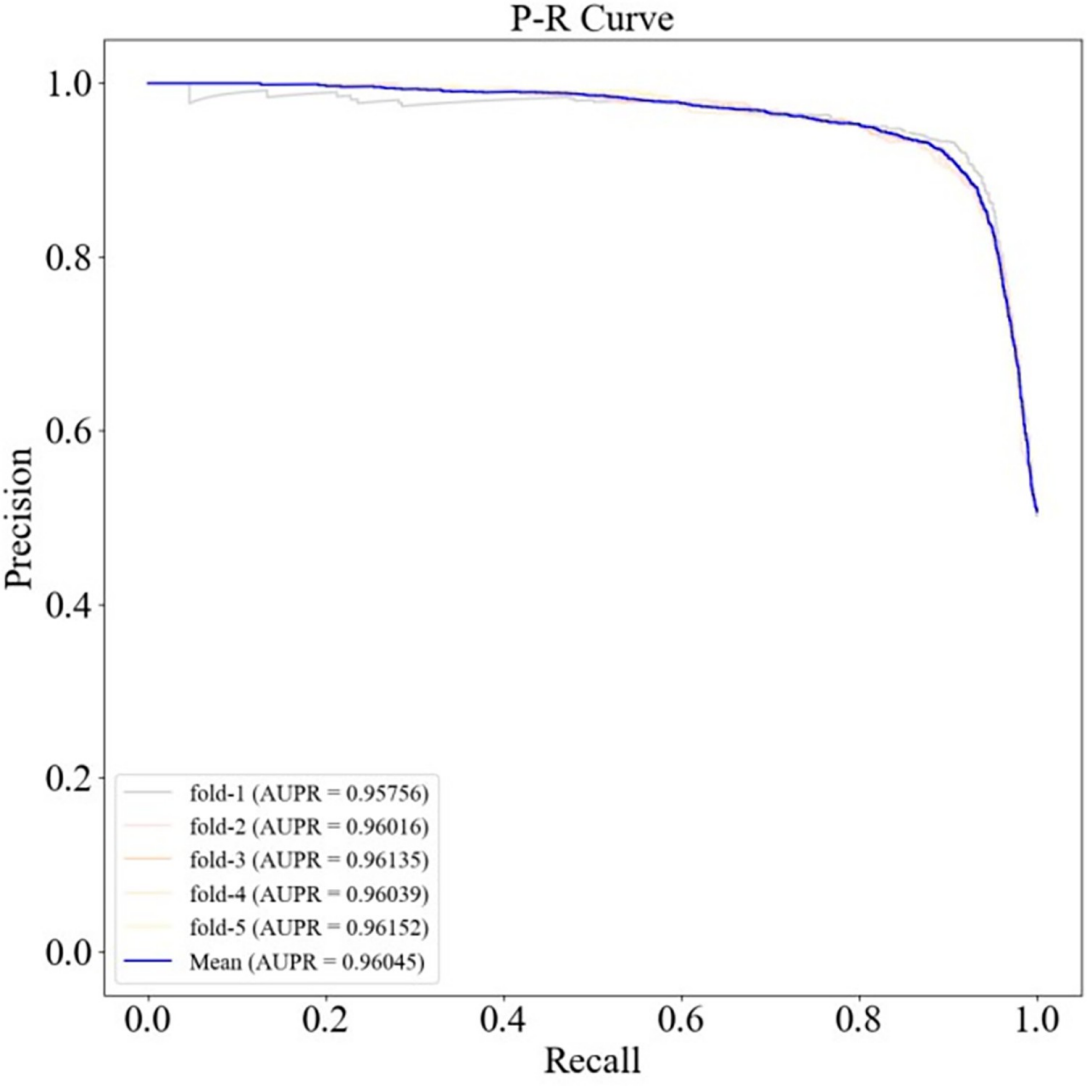
P-R curves under 5-fold cross-validation.

The ROC and PR curves of HGC-GAN under 10-fold cross-validation and the corresponding AUC and AUPR values are shown in Figs 4 and 5, respectively, with a mean AUC of 0.95548 and a mean AUPR of 0.95869. Additionally, 5-fold cross-validation was also performed, as shown in Figs 6 and 7, with a mean AUC of 0.95612 and a mean AUPR of 0.96054.

**Fig 8:**
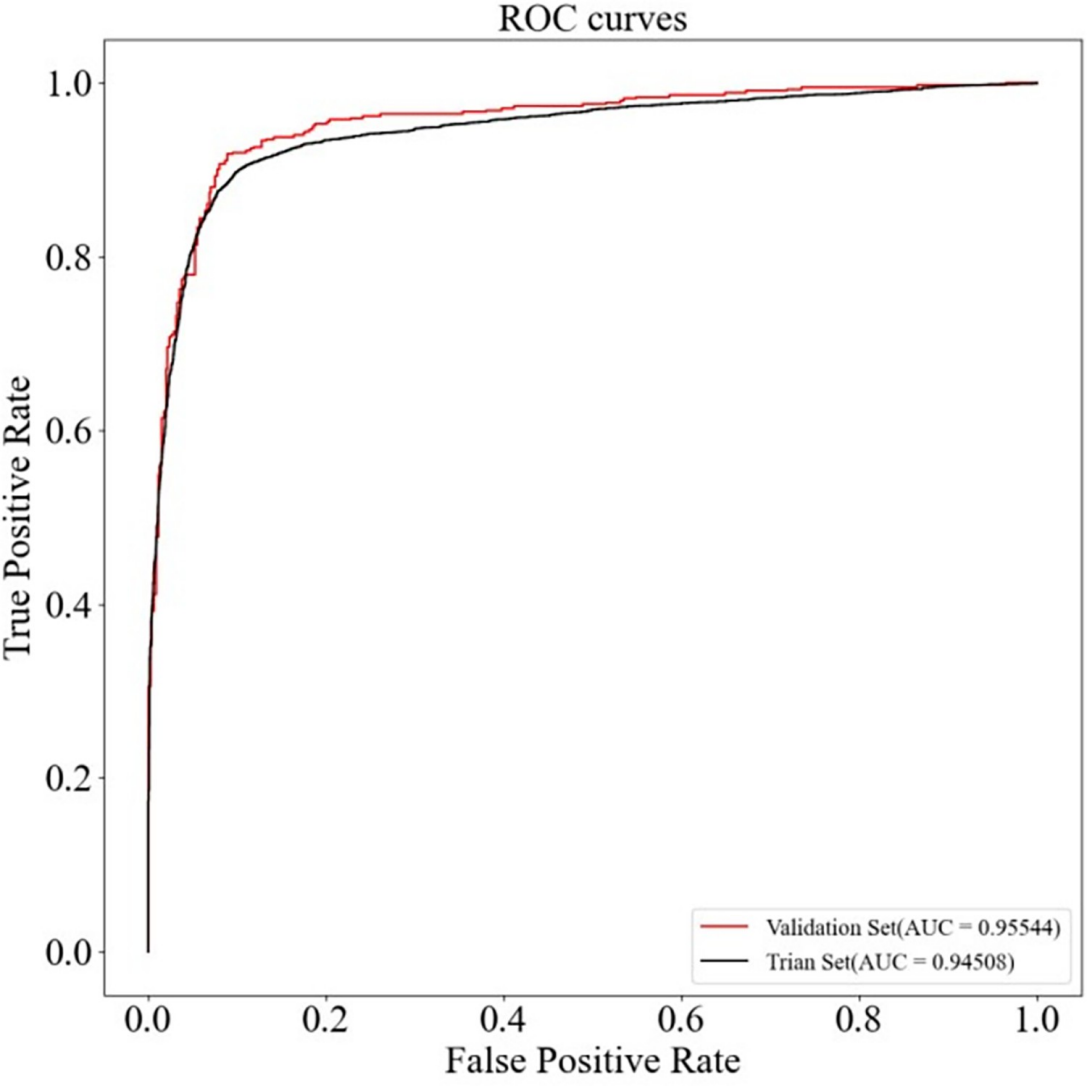
ROC curves of training and validation sets.

**Fig 9:**
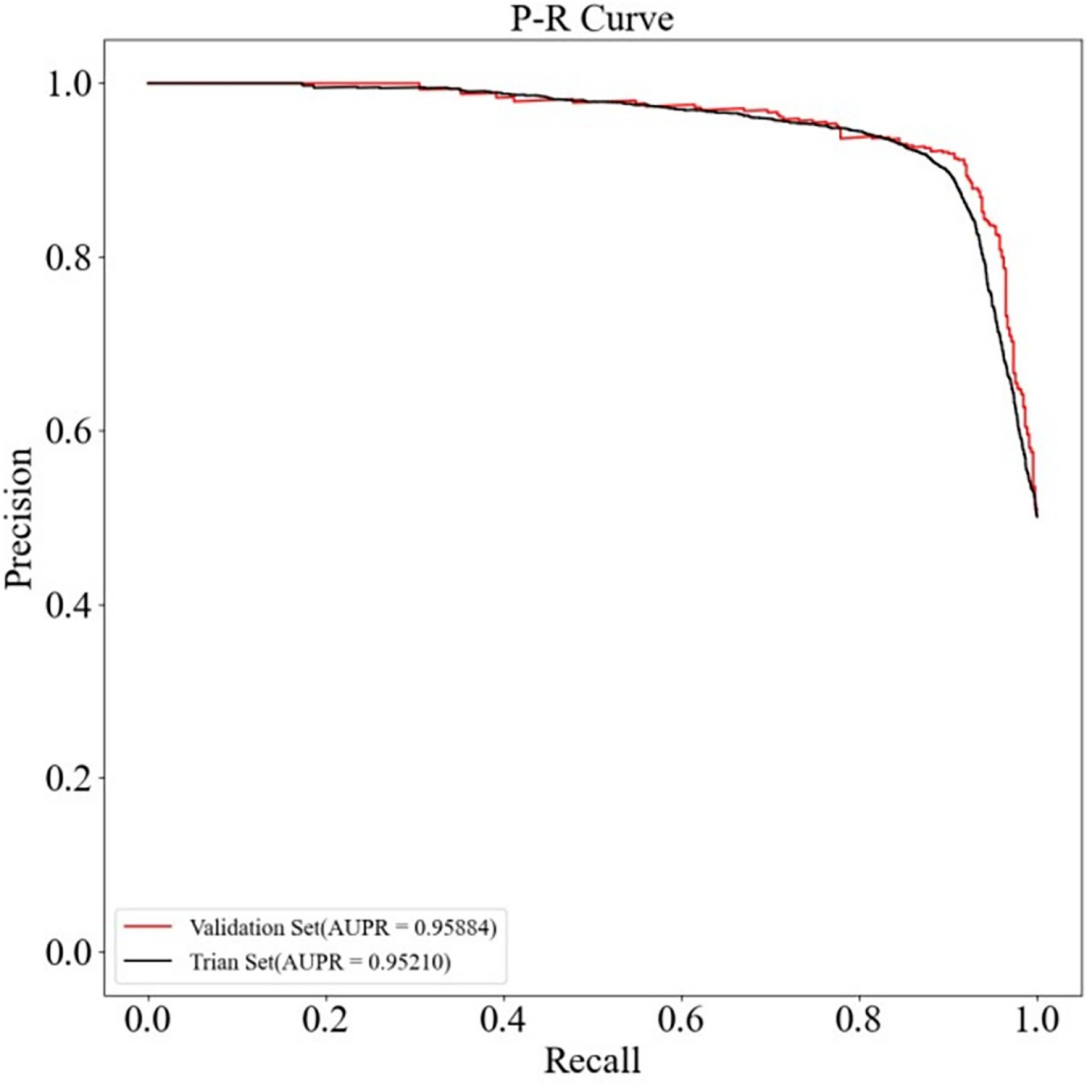
P-R curves of training and validation sets.

To confirm whether the experimental results of HGC-GAN were overfitted, one-tenth of the samples were further separated into an independent validation set, and the remaining samples were used for training HGC-GAN. The ROC and PR curves of the training and validation sets are shown in Figs 8 and 9. The AUC value of HGC-GAN reaches 0.95544, and AUPR reaches 0.95884 on the validation set, indicating that the excellent performance of the 10-fold cross-validation is not due to overfitting.

### Comparison of models

In HGC-GAN, the parameters were optimized using the RMSprop optimizer. The hyperparameter Epoch was set to 150, with the generator being trained five times and the discriminator being trained twice under each Epoch. The batch size was fixed at 32, and α was set to 0.1. All experiments were conducted under the same settings. To better assess the performance of our model, we conducted model comparison experiments on multiple datasets from various perspectives.

**Fig 10:**
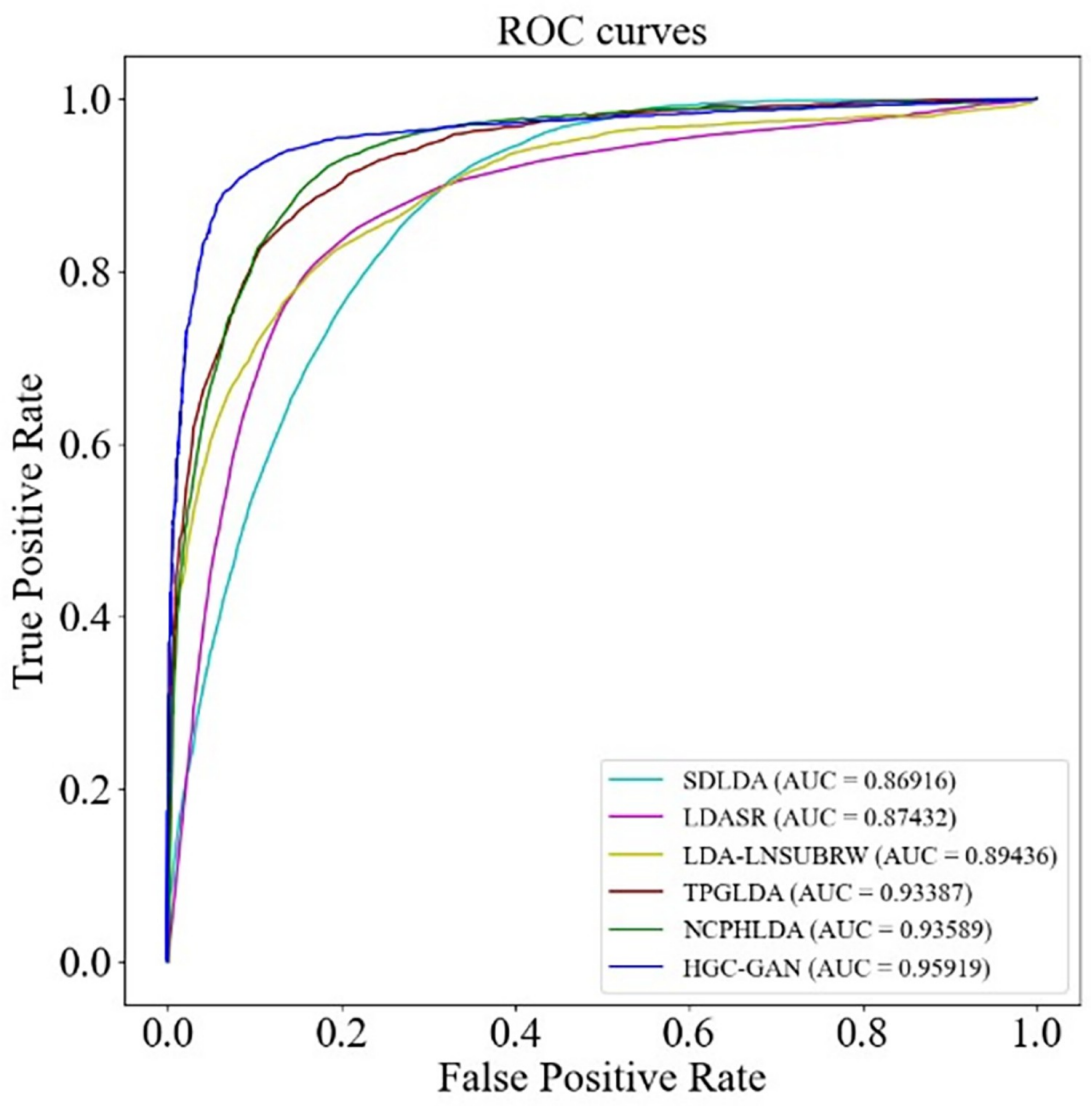
ROC curve for model comparison.

**Fig 11:**
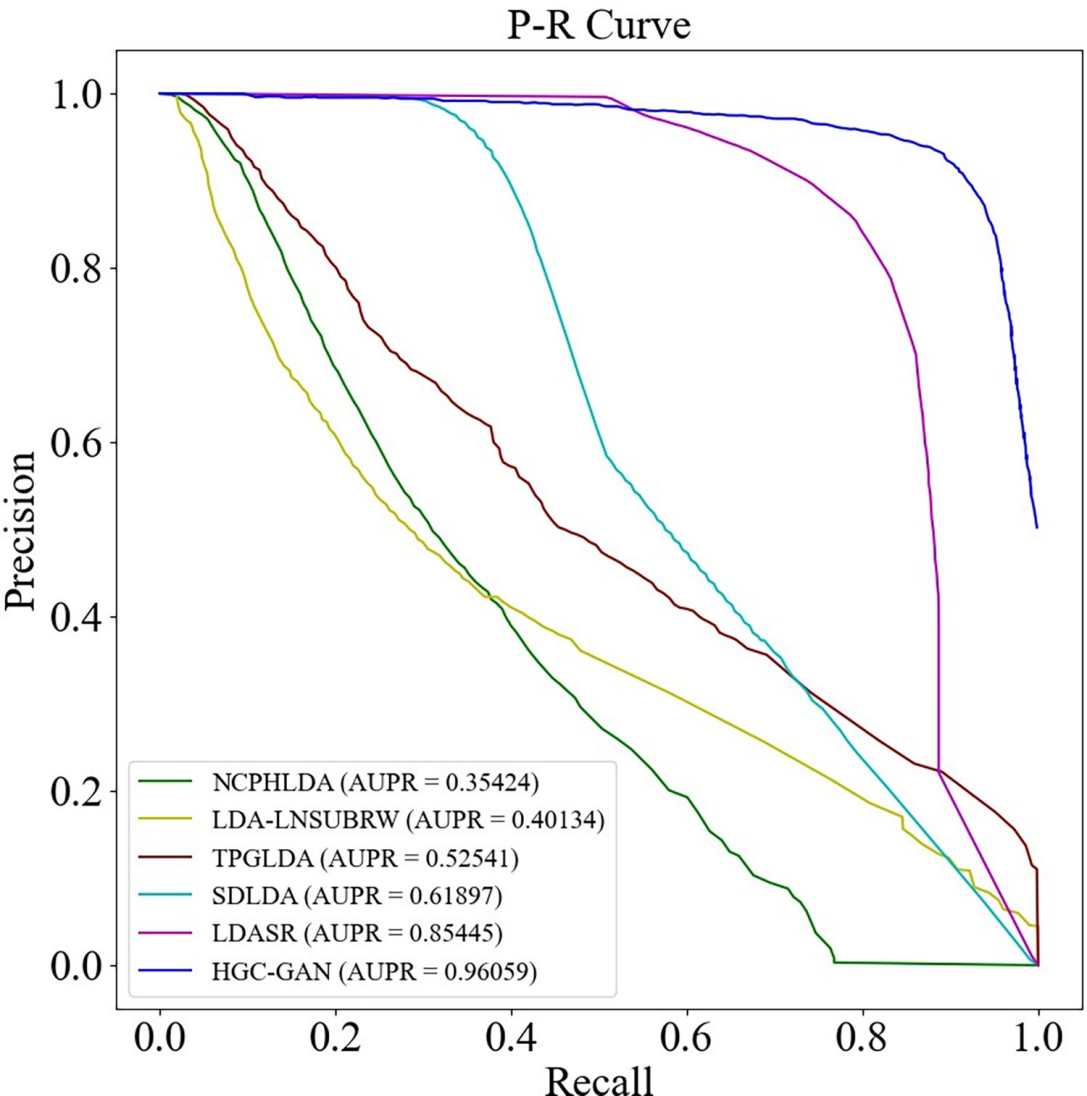
P-R curves for model comparison.

Firstly, we evaluated the performance of HGC-GAN on Dataset 1. We compared it with five state-of-the-art methods: SDLDA [25], LDASR [26], LDA-LNSUBRW [27], TPGLDA [28], and NCPHLDA [29]. All models were compared under the same experimental setup. The ROC and PR curves under 10-fold cross-validation and the AUC and AUPR values are shown in Figs 10 and 11, respectively.

The results demonstrated that HGC-GAN outperformed the other five state-of-the-art methods, achieving the highest AUC and AUPR values. The AUC of HGC-GAN was 9.003%, 8.487%, 6.483%, 2.532%, and 2.33% higher than SDLDA, LDASR, LDA-LNSUBRW, TPGLDA, and NCPHLDA, respectively. Correspondingly, AUPR was higher by 34.162%, 10.614%, 55.925%, 43.518%, and 60.635%. The performance of other methods was limited due to the sparsity of lncRNA-disease association. The superior performance of HGC-GAN can be attributed to the effective aggregation of association information and node information by the HGCN layer, while the sparsity issues of the data and the extreme imbalance between positive and negative samples are effectively mitigated by the use of the generative adversarial part.

Furthermore, to demonstrate the effectiveness of the HGC-GAN approach, we conducted comparative experiments on Dataset 2, comparing it with other GAN-based models and several state-of-the-art models. GANLDA [30] employs a graph attention network to extract valuable information from the features of lncRNA and diseases for the prediction of their associations. BiGAN [31] is a lncRNA-disease association prediction method based on a bidirectional generative adversarial network. gGATLDA [32] utilizes a graph-level Graph Attention Network (GAT) and Graph Neural Network (GNN) to predict LDAs. GAMCLDA [33] also makes use of graph convolutional networks (GCNs) to learn node features for association prediction. SIMCLDA [34] is a method for predicting potential LDAs through inductive matrix completion. MFLDA [35] fuses different heterogeneous data sources and predicts new associations using matrix factorization techniques. These models were evaluated under the same experimental conditions using ten-fold cross-validation, and the experimental results are presented in Table 2.

**Table 2:** AUC values for model comparison on dataset 3.

Model	HGC-GAN	GANLDA	BiGAN	gGATLDA	GAMCLDA	SIMCLDA	MFLDA
AUC	0.9310	0.8834	0.8930	**0.9867**	0.9071	0.8433	0.8217
AUPR	**0.9397**	0.0284	0.8851	0.9310	0.0378	0.8824	0.8720

The experimental results show that HGC-GAN exhibits higher accuracy in both AUC and AUPR compared to other GAN-based and state-of-the-art models. In terms of AUC, HGC-GAN is slightly lower than gGATLDA but higher than other state-of-the-art models, and in terms of AUPR, HGC-GAN outperforms all the models.

Finally, on Dataset 4, we compared HGC-GAN with three other models that also utilize heterogeneous data and are based on heterogeneous networks. RWRHLD [36] prioritizes candidate lncRNA-disease associations by integrating three networks (miRNA-associated lncRNA-lncRNA crosstalk network, disease-disease similarity network, and known lncRNA-disease association network) into a heterogeneous network and implementing a random walk with restart on this heterogeneous network. RWRlncD [37] infers novel lncRNA-disease associations based on a random walk model of a lncRNA functional similarity network. Methods [23] constructed a tripartite network by integrating heterogeneous data: containing known topological interactions of lncRNA-disease, lncRNA-microRNA, and microRNA-disease, and then predicting lncRNA-disease associations using network topological similarities derived from deep mining of the heterogeneous network. These models were all evaluated under the same experimental conditions. The experimental results are presented in Table 3.

**Table 3:** AUC values for model comparison on dataset 4.

Model	HGC-GAN	RWRHLD	RWRlncD	[23]
AUC	**0.9505**	0.9231	0.5432	0.9316

The experimental results show that the HGC-GAN method significantly outperforms the other three models, which are also based on heterogeneous data and heterogeneous networks, in predicting lncRNA-disease associations.

All the comparison experiments show that HGC-GAN demonstrates excellent lncRNA-disease prediction ability on datasets of different sizes and sparsity. Meanwhile, by comparing with other state-of-the-art models, including those that also apply GAN, heterogeneous data (multiple Linked Data), and heterogeneous networks, HGC-GAN shows higher performance. This achievement not only further validates the effectiveness of the HGC-GAN method, but also highlights its great potential for application in the in-depth study of lncRNA-disease association mechanisms, and assisting in disease diagnosis and treatment.

### Robustness and efficacy of models

**Fig 12:**
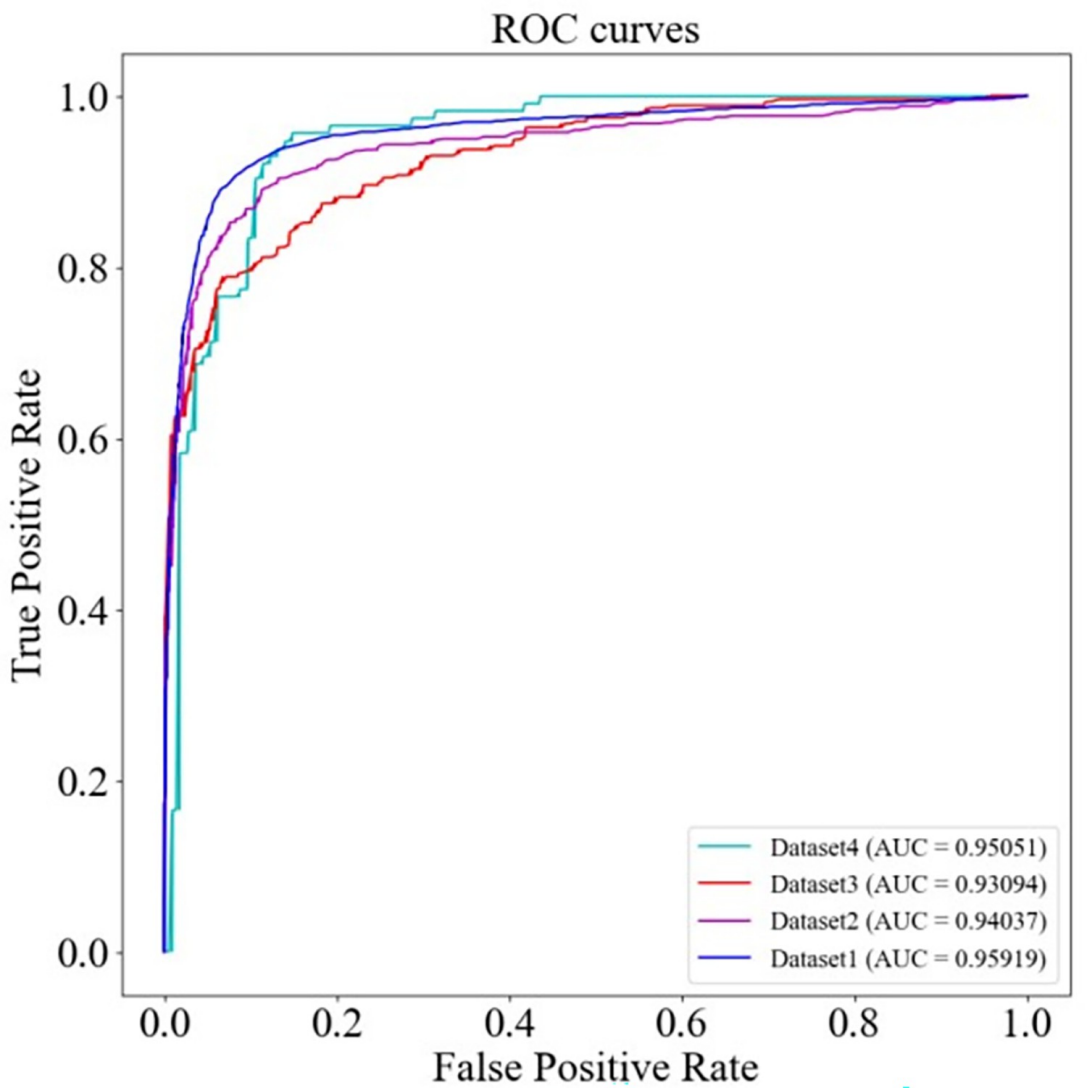
HGC-GAN AUC on different datasets.

**Fig 13:**
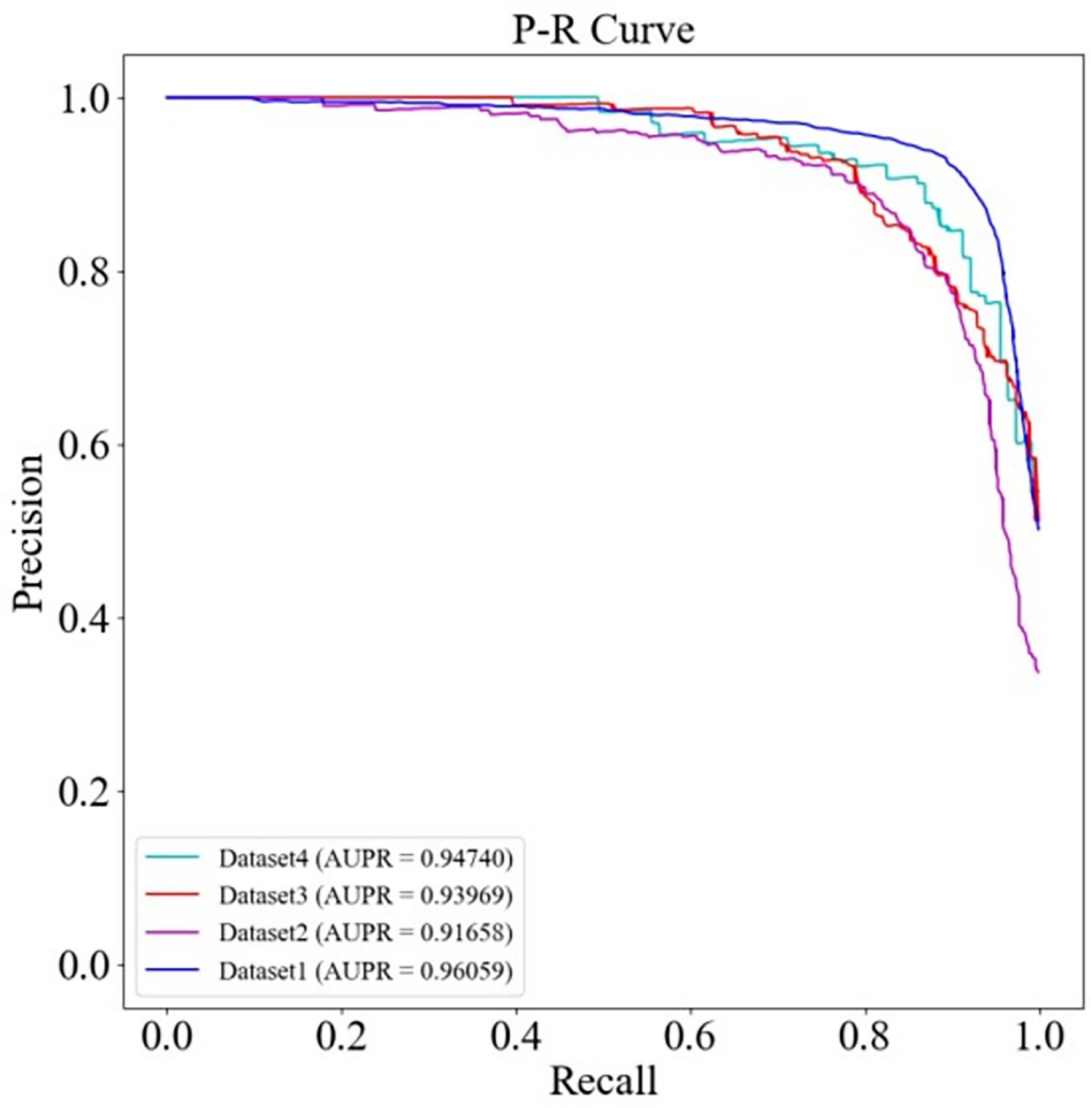
HGC-GAN AUPR on different datasets.

To validate the stable performance of HGC-GAN, it was applied to other datasets of different sizes that have been used in a number of other lncRNA-disease association prediction models. All experiments were based on 10-fold cross-validation, and the ROC and PR curves of HGC-GAN on these datasets are plotted in Figs 12 and 13, respectively.

The experimental results demonstrate that HGC-GAN performs well on all datasets and achieves excellent AUC and AUPR values, indicating its flexibility in handling data of different sizes. The generative adversarial networks’ adversarial learning effectively avoids the bias problem in most similarity-based computational methods caused by over-reliance on known associations, enabling HGC-GAN to deliver consistent performance on datasets.

### Explaining sequence features

In this section, the impact of lncRNA sequence features on the model was explained using the interpretable method LIME. Firstly, a random lncRNA-CASC9 was selected as the explanatory sample, which has been confirmed to have regulatory effects in various cancers [38], and its lncRNA sequence information (NCBI Reference Sequence: NR_103848.1, length 1471) was retrieved from NCBI. Then, the K-mer (K = 3) method was employed for feature extraction from the sequence, as described in the "Sequence Feature Extraction by K-mer" section, resulting in a 64-dimensional feature representation of the sequence. To explain the contribution of each feature dimension to the model’s decision, the LIME method was used to generate approximately 499 random samples based on this sample’s features. These 500 samples were then added to the LIME explainer and input to the HGC-GAN for prediction to assess the contribution level of the features. Through model predictions, the disease with the highest associated probability with CASC9 was identified as Acute Kidney Failure, and this association prediction has been confirmed [39]. Therefore, we conducted experimental analysis on the association between CASC9 and Acute Kidney Failure.

**Fig 14:**
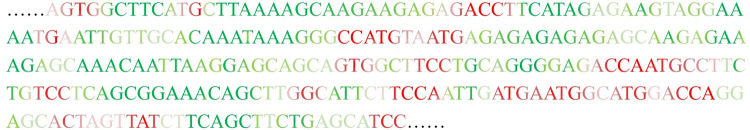
Heatmap of the CASC9 sequence.

**Fig 15:**
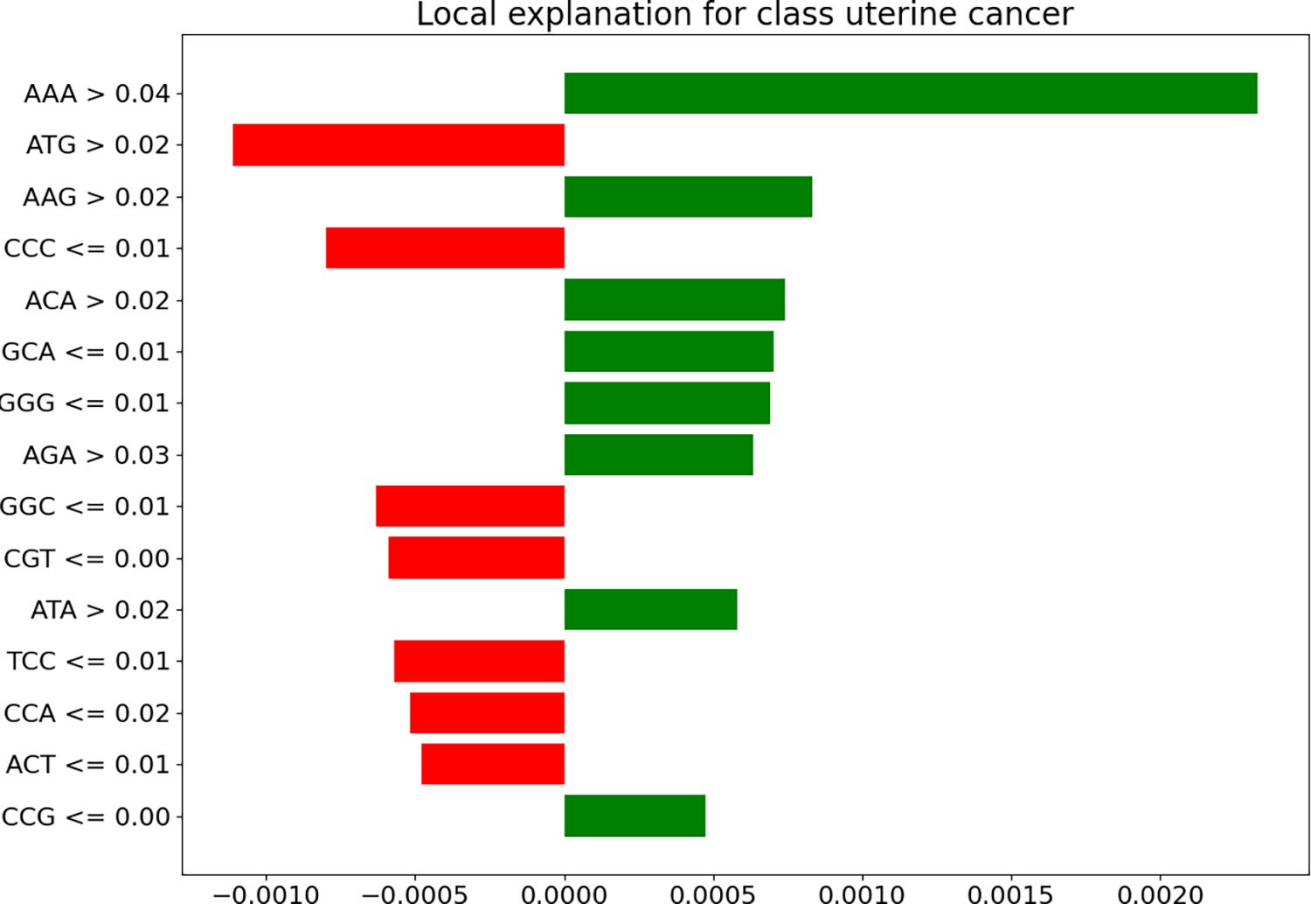
Weighting of nucleotide fragments.

We extracted a continuous nucleotide sequence from CASC9 and generated a heatmap based on the different nucleotide segments’ contributions to the model predictions, as shown in Fig 14. Green nucleotides represent contributions that support the association, while red nucleotides represent contributions that negate the association, with darker colors indicating greater contributions. We further explained the feature importance of the nucleotide segments, and the experimental results are displayed in Fig 15. In the figure, the green portion represents the features and their weights that support the association, while the red portion represents the features and their weights that do not support the association. For example, the portion of the AAA nucleotide segment with feature values greater than 0.04 has the greatest impact on the model’s decision and supports the association between CASC9 and Acute Kidney Failure. Conversely, the portion of the CCA nucleotide segment with feature values less than 0.02 largely does not support this association, indicating that different K-mer nucleotide segments have varying degrees of influence on the model’s decision. Through the analysis of the sequence heatmap and the K-mer contribution vectors, we found that the statistical results are consistent with the predictive results. The support contribution to the CASC9-Acute Kidney Failure association within the CASC9 sequence is greater than the negating contribution.

**Fig 16:**
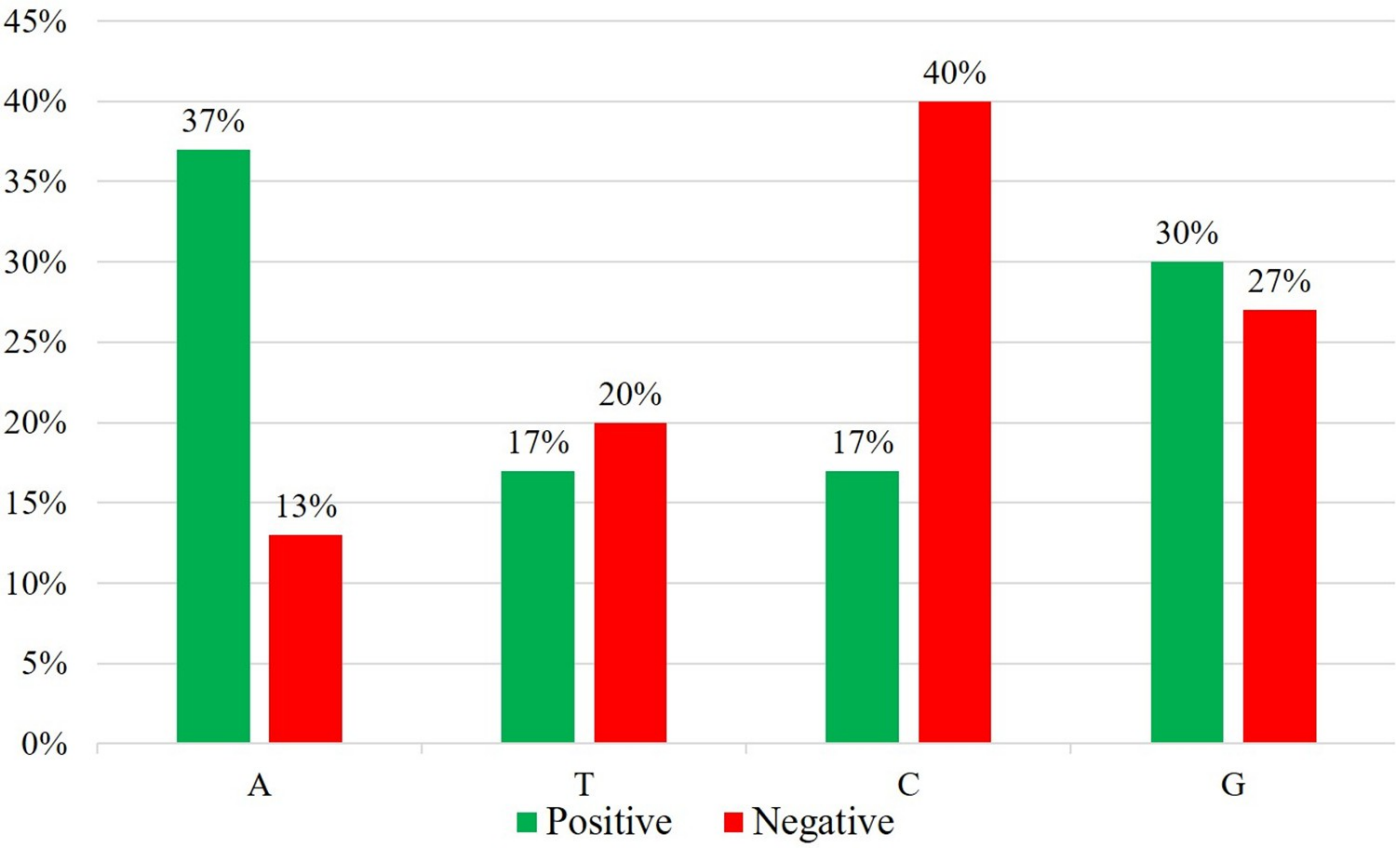
Nucleotides in positive and negative contributions.

To further investigate the K-mer contribution vectors, nucleotide combinations were sorted based on their positive and negative contribution scores. The top 10 nucleotide combinations with the highest positive contribution scores are AAA, GGG, GCG, ATA, TCA, CGG, ACA, CTG, ATT, and AAG. The top 10 nucleotide combinations with the highest negative contribution scores are CCA, CCC, TAT, TCC, CGC, ACC, ATG, GTG, GGC, and GGT. We calculated the proportion of the four nucleotides A, T, C, and G in both the top ten positive and negative contribution nucleotide combinations, as shown in Fig 16. We found that in the top ten positive contribution combinations, the combinations are primarily composed of adenine (A) and guanine (G), while in the top ten negative contribution combinations, the combinations are mainly composed of cytosine (C) and guanine (G). Therefore, in the prediction of the CASC9-Acute Kidney Failure association, nucleotide combinations with a high proportion of adenine and guanine support the association, while combinations with a high proportion of cytosine and guanine do not support the association.

### Ablation study

**Fig 17:**
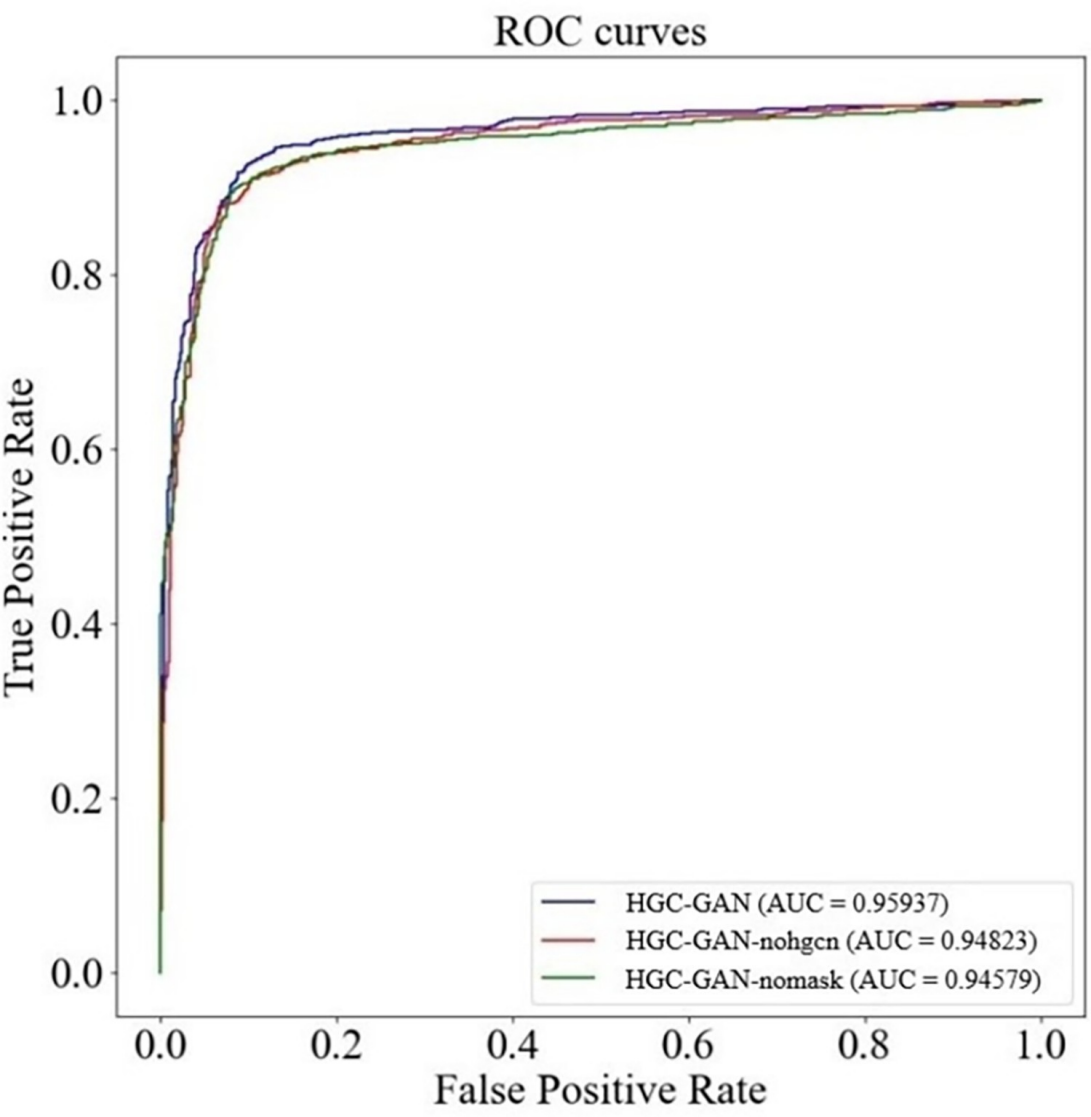
Ablative study of HGC-GAN.

To demonstrate the necessity of each component of HGC-GAN, we removed the HGCN component and the filter component separately, and calculated the model’s performance under the same experimental settings. Dataset 2 contains sequence information of lncRNAs, and aggregation of the information to embed potential features of biomolecules is achieved through HGCN. Therefore, we conducted this study on Dataset 2. The results are presented in Fig 17, with an AUC of 0.94823 when no HGCN was used for learning embedding, 0.94579 when the filter term was not used, and 0.95937 when the full HGC-GAN was used. The experimental results indicate that the model achieves the best performance when all network components are used. These results also demonstrate that the high-quality embedding representation obtained by the HGCN can effectively aggregate the neighborhood information of the graph and embed the characteristic information of the biomolecule itself, thereby improving the ability to predict potential associations.

### Effect of propagation layer

**Fig 18:**
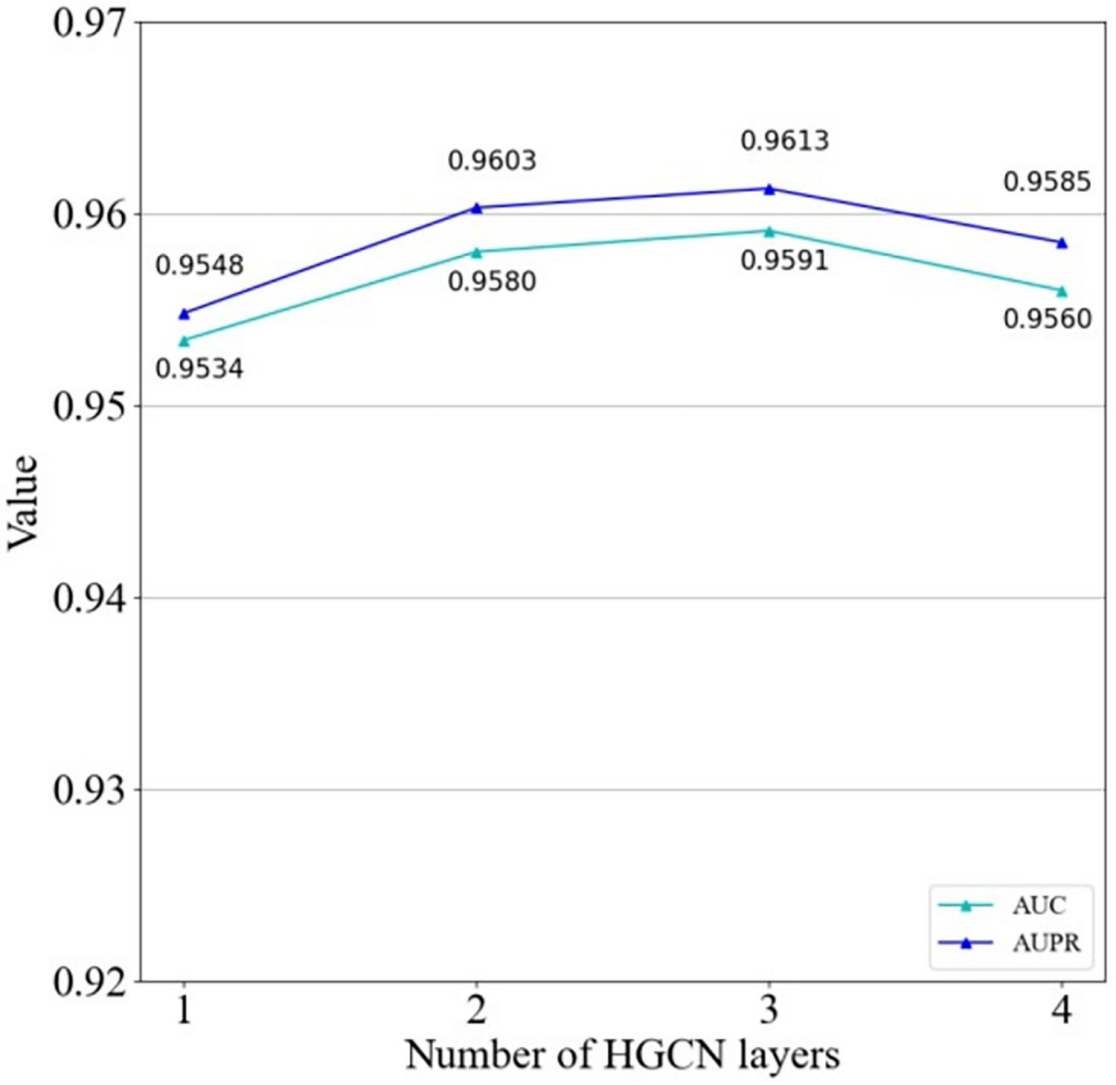
Effect of different layers on the model.

In order to evaluate the effectiveness of different propagation layers in the HGCN, different propagation layers were tested in the range of 1–4 with other parameters set to optimal. Fig 18 shows that the three-layer model is optimal, with the highest AUC and AUPR values of 0.9591 and 0.9613. The three-layer model outperformed the two- and one-layer model, while the four-layer resulted in an overly complex model with overly smooth node embeddings.

### Effect of embedding size

**Fig 19:**
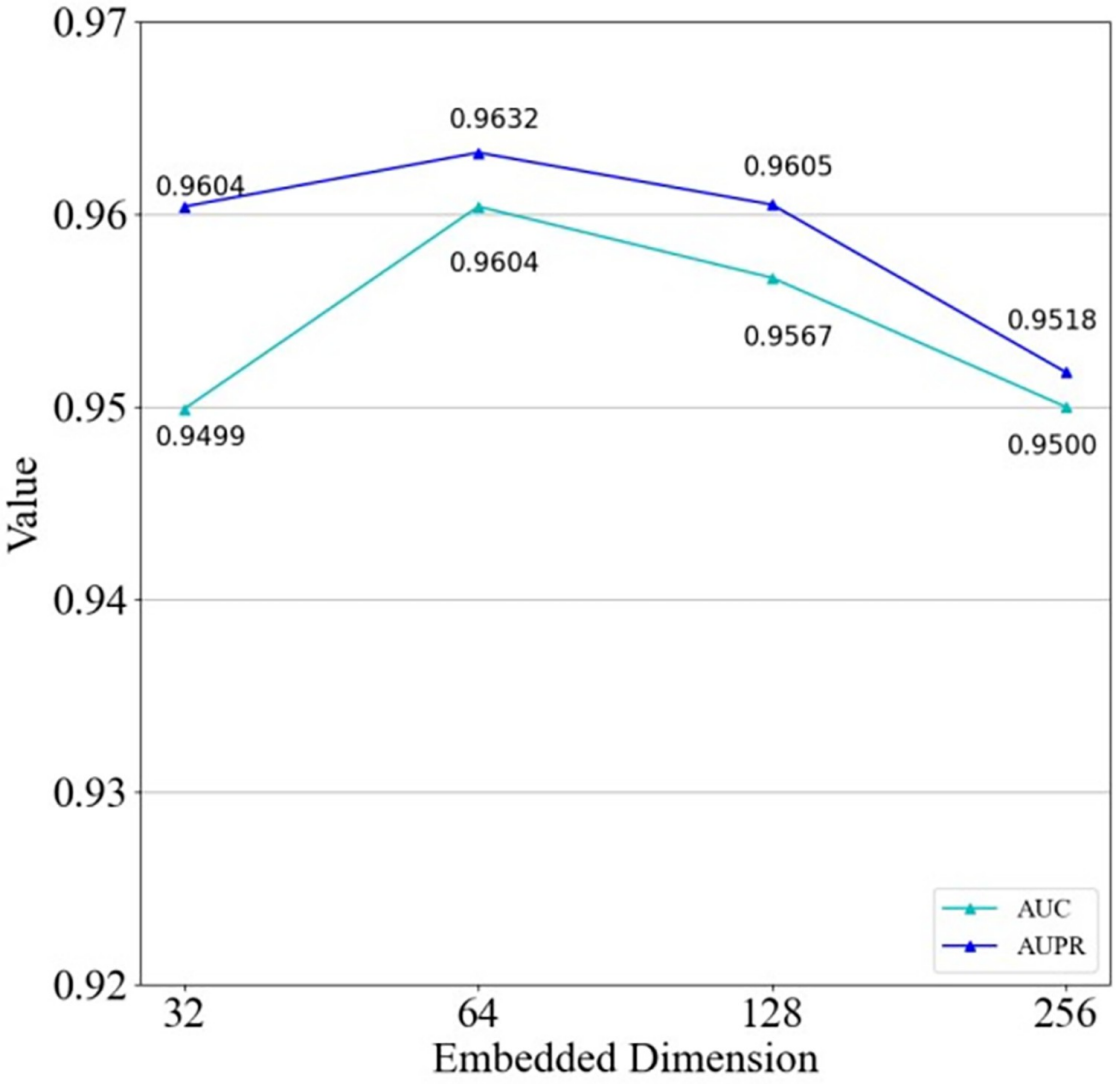
Effect of embedding size on the model.

The embedding size plays an important role in HGC-GAN and can directly affect the performance of the model. In the experiments, different embedding dimensions d (i.e., 32, 64, 128, 256) are set in the propagation layer and the prediction performance is evaluated under different settings. As shown in Fig 19, the best performance was achieved when the embedding dimension d = 64, with the highest AUC and AUPR values of 0.9604 and 0.9632. Therefore, a three-layer (64-64-64) network structure was chosen in the HGCN. A four-layer structure (256-512-1024-861) was designed to obtain the association score of lncRNA-disease in G-Network section, and a four-layer structure (1024-128-16-1) was designed to obtain the authenticity score of the input data in the D-Network section.

### Case study

To evaluate the ability of HGC-GAN to predict unknown associations based on sequence information of new lncRNAs, a case study was conducted. Firstly, a new lncRNA, linc00152 (also known as C2orf59, NCRNA00152), was selected from the NCBI database. The sequence information for this lncRNA was collected at a length of 852 bp, and it was ensured that this lncRNA did not participate in any training and testing parts. The K-mer feature extraction was then performed on the linc00152 sequence, and the extracted features were added to the constructed heterogeneous network (LMDN), using the HGCN part of the HGC-GAN model, to aggregate the information for linc00152, thus obtaining the updated node features. Since we do not know the association information of the new lncRNAs, we concatenate a 1×*d* (*d* denotes the number of diseases) all-0 matrix with the node features, and then output the association scores of linc00152 with each disease using the G-Network part of the HGC-GAN model, and sort all predicted nodes based on the association prediction scores. Finally, the predicted associations were confirmed by searching the most recent published literature.

Studies have demonstrated that linc00152 plays a regulatory role in many human life activities and is also involved in several cancers [40,41,42]. To validate the predictive performance of HGC-GAN, we selected linc00152 (also known as C2orf59, CYTOR, NCRNA00152) and evaluated its associations with diseases. The Lnc2CancerV3.0 database [20] includes 27 diseases associated with linc00152. Among the top 20 candidate associations with the highest prediction scores from HGC-GAN (Table 4), 19 have been confirmed to be related. Notably, the association of linc00152 with non-small cell lung cancer (also known as non-small cell lung carcinoma) and stomach cancer was not included in the Lnc2CancerV3.0 database, but HGC-GAN successfully predicted both associations, which have been confirmed [43,44]. Research [43] demonstrated that CYTOR could directly interact with miR-195 and enhance its target gene expression. CYTOR played an oncogenic role in the progression of non-small cell lung cancer (NSCLC) by sequestering miR-195. Research [44] indicated that LINC00152, acting as a competing endogenous RNA (ceRNA) of HMGA1, exhibited similar functions to HMGA1. It was found that LINC00152 could regulate the cell cycle and facilitate the proliferation of gastric cancer (GC) cells. Data from The Cancer Genome Atlas (TCGA) database suggested that LINC00152 might serve as a potential diagnostic marker for gastric cancer.

**Table 4:** Top 20 diseases associated with linc00152.

Rank	Disease	PMID	Rank	Disease	PMID
1	hepatocellular carcinoma	30779070	11	stomach cancer	35014092
2	colorectal cancer	31144988	12	bladder cancer	29988223
3	breast cancer	31894257	13	cervical cancer	31114990
4	gastric cancer	32210577	14	non-small cell lung carcinoma	30487160
5	lung adenocarcinoma	28109288	15	glioblastoma	29991527
6	prostate cancer	Unconfirmed	16	esophageal squamous cell carcinoma	30784933
7	glioma	27923049	17	colon cancer	29606502
8	non small cell lung cancer	30487160	18	papillary thyroid cancer	30146756
9	ovarian cancer	31799647	19	acute myeloid leukemia	30707636
10	lung cancer	28592840	20	pancreatic cancer	25910082

In addition to known associations, HGC-GAN can predict disease-related lncRNAs for diseases. Breast cancer, the most common cancer in women after skin cancer, was chosen to validate the performance of HGC-GAN. Studies have shown that lncRNAs play an important role in the development of breast cancer [45,46]. Table 5 shows the top 20 candidate lncRNAs associated with breast cancer with the highest HGC-GAN prediction scores. None of these predicted potential associations were included in the LncRNADiseaseV2.0 and Lnc2CancerV3.0 databases, indicating that the predictions were potentially unknown associations. It is noteworthy that HGC-GAN successfully predicted 17 out of the top 20 highest scoring candidate associations, and these associations have been confirmed by recent studies.

**Table 5:** Top 20 lncRNAs associated with breast cancer.

Rank	lncRNA	PMID	Rank	lncRNA	PMID
1	afap1-as1	32376943	11	flvcr1-as1	32518523
2	snhg11	34257707	12	pcat7	33401925
3	foxd2-as1	34873418	13	rmrp	35143945
4	cps1-it1	Unconfirmed	14	hoxb-as1	35273635
5	fer114	35331091	15	mir31hg	34076993
6	hnfla-as1	33603481	16	dleu2	33987091
7	mir100hg	30042378	17	slc2a1-as1	Unconfirmed
8	myd88	30066873	18	linc00596	Unconfirmed
9	mir210hg	35346372	19	neat1_2	35581633
10	igf2-as	33175607	20	linc00261	33274565

These results demonstrate that HGC-GAN has good predictive ability and shows excellent performance in association prediction of new lncRNAs, including previously unknown associations.

## Conclusion

An increasing number of studies have shown that lncRNAs play crucial roles in the development of cancers, cardiovascular diseases, and neurological disorders. However, many existing computational methods are limited in their ability to extract multi-node association information and fuse node features, relying solely on association information for prediction. This not only limits the richness of biomolecular information but also significantly reduces the confidence of prediction results. Furthermore, the sparsity of lncRNA-disease association data limits the performance of many computational methods.

To address these challenges, this study proposes a novel lncRNA-disease association prediction model, HGC-GAN, which combines heterogeneous graph neural networks and generative adversarial networks. By using heterogeneous graph neural networks, HGC-GAN can effectively aggregate information of neighbors in the graph and embed it into the feature information of biomolecules themselves, avoiding data homogeneity and making full use of the biomolecule’s features to improve the confidence of the prediction results. In addition, the use of generative adversarial networks can effectively alleviate the problem of data sparsity and extreme imbalance between positive and negative samples of data, and avoids the bias problem caused by over-reliance on known associations in most similarity-based computational methods through adversarial learning.

To validate the effectiveness of HGC-GAN, a 10-fold cross-validation-based comparison was conducted with five state-of-the-art methods, including SDLDA, LDASR, LDA-LNSUBRW, TPGLDA, and NCPHLDA. The excellent results of the case study suggest that HGC-GAN is a useful tool for predicting potential lncRNA-disease associations. Future research will integrate more biological data and information on the diversity of biomolecules themselves to explore more ways to extract high-quality node embeddings for predicting unknown associations.

**Key Points**

- Evidence supports the important role of lncRNAs in various cancers and diseases, highlighting the need for computational models to predict potential lncRNA-disease associations.
- Four datasets of different sizes were constructed by collecting multiple association data and lncRNA sequence information from public datasets and papers, and heterogeneous graphs were constructed based on these datasets. All data and codes are available for further study.
- A new computational method, HGC-GAN, was proposed by combining HGCN and GAN to predict potential lncRNA-disease associations. It overcomes the problems of sparse association information and the lack of negative samples, effectively improving the reliability and accuracy of prediction results while utilizing the richness of biomolecule information.
- Experimental results demonstrate that HGC-GAN outperforms other advanced methods. The case study also shows that HGC-GAN can predict potential associations of novel lncRNAs without any known lncRNA-disease associations.
